# Quantitative Magnetic Resonance Imaging in Limb-Girdle Muscular Dystrophy 2I: A Multinational Cross-Sectional Study

**DOI:** 10.1371/journal.pone.0090377

**Published:** 2014-02-28

**Authors:** Tracey A. Willis, Kieren G. Hollingsworth, Anna Coombs, Marie-Louise Sveen, Soren Andersen, Tanya Stojkovic, Michelle Eagle, Anna Mayhew, Paulo Loureiro de Sousa, Liz Dewar, Jasper M. Morrow, Christopher D. J. Sinclair, John S. Thornton, Kate Bushby, Hanns Lochmuller, Michael G. Hanna, Jean-Yves Hogrel, Pierre G. Carlier, John Vissing, Volker Straub

**Affiliations:** 1 Institute of Genetic Medicine, Newcastle University, Newcastle upon Tyne, United Kingdom; 2 The Robert Jones and Agnes Hunt Orthopaedic Hospital, Oswestry, Shropshire, United Kingdom; 3 Newcastle Magnetic Resonance Centre, Institute of Cellular Medicine, Newcastle University, Newcastle upon Tyne, United Kingdom; 4 Neuromuscular Research Unit, Department of Neurology, Rigshospitalet, University of Copenhagen, Copenhagen, Denmark; 5 Centre de Reference des Maladies Neuromusculaires Paris Est, Institut de Myologie Groupe Hospitalier Pitié-Salpêtrière, Paris, France; 6 Institut de Myologie Groupe Hospitalier Pitié-Salpêtrière, Paris, France; 7 MRC Centre for Neuromuscular Diseases, Department of Molecular Neurosciences, UCL Institute of Neurology, London, United Kingdom; Stem Cell Research Institute, Belgium

## Abstract

We conducted a prospective multinational study of muscle pathology using magnetic resonance imaging (MRI) in patients with limb-girdle muscular dystrophy 2I (LGMD2I). Thirty eight adult ambulant LGMD2I patients (19 male; 19 female) with genetically identical mutations (c.826C>A) in the fukutin-related protein (*FKRP*) gene were recruited. In each patient, T1-weighted (T_1_w) imaging was assessed by qualitative grading for 15 individual lower limb muscles and quantitative Dixon imaging was analysed on 14 individual lower limb muscles by region of interest analysis. We described the pattern and appearance of muscle pathology and gender differences, not previously reported for LGMD2I. Diffuse fat infiltration of the gastrocnemii muscles was demonstrated in females, whereas in males fat infiltration was more prominent in the medial than the lateral gastrocnemius (p = 0.05). In the anterior thigh of males, in contrast to females, median fat infiltration in the vastus medialis muscle (45.7%) exceeded that in the vastus lateralis muscle (11.2%) (p<0.005). MRI is non-invasive, objective and does not rely on patient effort compared to clinical and physical measures that are currently employed. We demonstrated (i) that the quantitative Dixon technique is an objective quantitative marker of disease and (ii) new observations of gender specific patterns of muscle involvement in LGMD2I.

## Introduction

Limb-girdle muscular dystrophy 2I (LGMD2I) is an autosomal recessive disease caused most frequently by a homozygous founder mutation (c.826C>A) in the fukutin-related protein gene (*FKRP*) [1], [2]. This condition is clinically heterogeneous with varying presentations, including age of onset, rate of progression and degree of severity. At the mild end of the spectrum, patients can present with isolated asymptomatic hyperCKaemia in adulthood and at the severe end of the spectrum, with a more progressive disease resembling Duchenne muscular dystrophy with an early onset in childhood. Respiratory and cardiac involvement is frequently seen in LGMD2I patients and can occur independently of the general muscle weakness [3]–[6].

While magnetic resonance imaging (MRI) can have a role in the diagnostic workup of patients with inherited muscle diseases [7]–[10], with MRI changes sometimes predating clinical/functional changes [11], this is less important than genetic profiling in the diagnosis of LGMD2I. However, quantitative fat imaging by Dixon MRI can provide information about the state of fat replacement in individual muscle groups as a measure of muscle pathology. This information will be useful for clinical trials, since for this purpose we must not study muscles that are too severely affected at baseline and unlikely to respond to treatment, nor those which are not involved at all and unlikely to deteriorate in the absence of treatment. To date, most MRI studies have used T1-weighted (T1w) imaging in small numbers of patients, grading the fat replacement on a 6-point visual scale. This is however, subjective and does not permit discrimination of small changes in muscle pathology.

In this study we have employed both qualitative T_1_w imaging and quantitative fat imaging using the Dixon technique, to directly compare these methods and describe the muscle involvement of the cohort. The quantitative Dixon technique separates the fat and the water components of the MR signal allowing the calculation of tissue fat fraction, which is observer independent [12]. In addition, it corrects for the inhomogeneity in magnetic and radiofrequency fields, which makes signal intensity on T_1_w images difficult to quantify (see Figures S1 and S2 in File S1 for illustration). In this work, we have described in detail both the patterns of affected muscles and their individual appearance on T_1_w images in patients with LGMD2I. We have presented quantitative analysis using the quantitative Dixon technique and have compared these figures with the T_1_w qualitative scores. This objective quantitative approach is vital for accurately defining the natural history of skeletal muscle involvement in LGMD2I.

## Materials and Methods

### Setting and study population

This study complied with the Declaration of Helsinki, and ethical approval for the Newcastle upon Tyne and London centers was provided by the Newcastle and North Tyneside Research Ethics Committee 2 (reference 09/H0907/29); the Regional Committee on Biomedical Ethics of Copenhagen (reference H-B-2009-061) provided approval for the Copenhagen centre; and the Local Ethics Committee CPP-Ile-de-France VI (reference 2009-A00808-49) approved the Paris center. Written consent was obtained from all participants. Thirty eight ambulant adults with a genetically confirmed diagnosis of LGMD2I (19M:19F age 18–64 years, mean ages of 41.7 years and 39.5 years, respectively, and disease duration 0–49 years) were recruited prospectively. We also recruited 8 healthy adult controls (5M: 3F age range 23–61 years, mean 40 years). Inclusion criteria were: identical genetic diagnosis with homozygous c.826C>A *FKRP* mutations, ambulant for more than 50 metres, no ventilator requirements and able to lie flat with no contraindications for MRI scanning. Data were acquired locally at the participating centres, using pre-agreed standardised MRI protocols and clinical and functional assessments scores with a standardised reporting method. Quality assurance was performed for all centres prior to the study.

### MRI techniques

The study consisted of (i) standard T_1_w imaging, allowing for whole muscle qualitative evaluation and (ii) quantitative fat imaging optimised for localised analysis of fat fraction. These protocols were defined for use on 3.0 T scanners (Philips Intera Achieva, Siemens TIM Trio) with surface-coil arrays for signal detection.

#### i. T_1_w imaging

T_1_w imaging was performed with a turbo spin echo sequence with the following parameters: Repetition Time/Echo Time (TR/TE) = 671/10 ms (Newcastle/Paris) or 16 ms (London) or 12 ms (Copenhagen), number of averages 2, acceleration factor 3, slice thickness 5 mm, interslice gap 10 mm, 256×192 matrix interpolated to 512×384. The field of view (FOV) was 410 mm using multiple stacks to cover both legs from the ankle to the pelvic crest. By specifying the sequence and repetition time, equivalent fat/water contrast in the image was achieved across the centres and this was verified in an acquisition from a healthy volunteer before the patient data collection began.

#### ii. Quantitative Dixon imaging

Spoiled gradient echo sequences were used. Protocol details varied slightly between sites. Newcastle/London: 3-point Dixon images acquired in 2D with TR/TE = 100/3.45,4.6,5.75 ms, flip angle  = 10 degrees, 10 slices of 10 mm slice thickness, 5 mm gap; Paris: 3D, 64 slices of 5 mm slice thickness with TR/TE = 10/2.75,3.95,5.15 ms, flip angle  = 3 degrees; Copenhagen: as per Paris, but with 36 slices per 3D acquisition, 2-point Dixon with correction for B_0_ inhomogeneity [13]. Optimal echo times were determined locally due to variation in precise B_o_ magnitude between scanners. Images were collected at mid-lower leg and mid-thigh. The central plane of acquisition was defined with respect to bone landmarks as follows: legs were positioned with the patella anterior; the lower leg images were centred by finding the broadest part of the lower leg muscle, and recording the distance from lower border of patella. Thigh images were centred by locating superior border of patella, and the anterior superior iliac spine, with centering 1/3 distance superior to the patella; the distance was recorded for follow-up scans. Each leg was imaged individually using 160×160 matrix interpolated to 256×256, FOV 200×200 mm. In Paris, it was possible to scan both legs together at the same resolution using FOV 448×244 mm. The data was analysed to produce separate fat and water images [13], [14], see Figure S2 in File S1. Quantitative fat fraction maps were produced by expressing the fat signal as a percentage of the total signal per voxel.

Due to the need to use different scanners for implementation of the Dixon method across the centres, two variations in the Dixon method were used [13], [14]. We ensured that the implementations did not give different quantitative results by (i) agreeing equivalent proton-density weighted protocols of a defined resolution, (ii) running quantitative acquisitions on phantoms of known water-fat composition with each implementation (by taking progressive slices through a water-fat boundary) for comparison and (iii) scanning control test subjects to ensure that equivalent regions of interest could be marked on the images across the four centres.

#### iii. Interpretation

The images were analysed by one consultant neurologist (TW). The T_1_w images were assessed on an individual muscle basis and graded according to the scale published by Mercuri et al. [15], [16]. The quantitative fat images were analysed by defining regions of interest (ROIs) in individual muscles on the separated water image at the midpoint of both the lower leg and thigh. The muscle groups analysed were, in the upper leg, the biceps femoris long head and short head, semitendinosus, semimembranosus, sartorius, gracilis, rectus femoris, vastus lateralis and vastus medialis muscles; in the lower leg, muscle groups analysed were the tibialis anterior, soleus, peroneus longus, and lateral and medial gastrocnemius muscles. To assess the objectivity of the measurements made, independent grading of T_1_w images and ROI assessment of quantitative Dixon images in all muscle groups in a randomly-selected subset of 8 patients (2 from each centre) was performed by KGH, an MR physicist with 6 years' experience of muscle imaging. Bland-Altman analysis was performed on the quantitative Dixon imaging results from the 14 muscle groups on each of the subjects to assess bias and reproducability [17], while the intraclass correlation coefficient was calculated for the gradings. The intrinsic repeatability of the technique and positioning was assessed on 6 control subjects by acquiring quantitative fat images twice in close succession, removing the subjects from the scanner and repositioning them. The same ROIs analysed in the patients were analyzed by the same observer (KGH).

### Functional testing

Functional tests were performed in the patients at the time of MRI to add information on clinical severity, with the 4 centres working to a standardised manual. These tests included myometry strength measurement using a hand held myometer (measuring hip flexion and extension, knee flexion and extension, ankle dorsiflexion and plantar flexion), the 6 minute walk distance (6MWD), the 10 metre walk, the timed chair rise, timed stair climb and timed stair descent (using 4 standard stairs with rails) and the ‘timed up and go’ (TUG) test. Forced vital capacity (FVC) was measured as the best of three attempts using a hand-held spirometer with the patient in a sitting and then in a supine position and related to the expected value for patient height.

### Statistical Methods

The Shapiro-Wilk test was used to demonstrate that the measured variables were not normally distributed. Non-parametric statistical tests were used for analyses using the SPSS version 17.0 software (IBM, USA). Spearman's rank correlation (coefficient reported as r_s_) was used to compare the Dixon quantitative fat analysis values and the qualitative scores. Comparisons of the fat fractions between the muscles were analysed using the Wilcoxon signed-rank test and between genders using the Mann-Whitney U test.

## Results

The patients represented the broad phenotypic spectrum that is commonly observed in patients with the common c.826C>A mutation in the *FKRP* gene. The spectrum ranged from asymptomatic patients to those who were almost non ambulant and just able to walk 50 m. This is detailed in Table 1, with onset of symptoms in childhood (less than 16 years) recorded in approximately half the study group (47%) with just over a quarter (26%) of the group developing symptoms aged 10 years or younger.

**Table 1:** Summary table of the clinical characteristics of the LGMD2I cohort (n = 38).

Patient	Sex	Age (years)	First symptom	Age First Symptom	Serum CK	Cardiomyopathy Y/N	Respiratory involvement Y/N
1	M	62	Flat feet, difficulty climbing stairs	45	2335	Yes	Yes
2	M	64	Poor running and falls	15	2471	Yes	Yes
3	F	58	Difficulty getting out of a chair and climbing stairs	43	n/a	Yes	No
4	M	46	Weight loss	23	1212	Yes	No
5	F	55	Difficulty climbing stairs and falls	41	n/a	No	No
6	M	21	Toe walker and slow at running	9	8050	Yes	No
7	F	25	Slow at running and odd gait when climbing stairs	9	2600	No	Yes
8	M	33	Raised CK detected when giving blood.	Early 20's	12000	No	No
9	M	28	Recurrent falls	24	3302	Yes	No
10	M	39	Poor at sport in school, unable to climb ropes.	10	n/a	Yes	No
11	M	54	Poor running and myoglobinuria	Late 40's	1000	Yes	No
12	M	37	Recurrent falls	Early teens	n/a	No	No
13	F	41	Slow at running and unable to climb school wall	10	222	No	No
14	F	46	Difficulty climbing stairs	22	856	No	Yes
15	F	46	Difficulty climbing stairs after first pregnancy	31	n/a	No	No
16	F	29	Pain in thighs and difficulty climbing stairs	12	13414	No	No
17	F	45	Difficulty climbing stairs	23	421	No	No
18	M	50	Difficulty climbing stairs	37	4950	Yes	No
19	M	30	Pain in buttocks and thighs	15	7545	No	No
20	F	27	Difficulty descending stairs	17	23858	No	No
21	F	30	Pain and cramps in legs	10	n/a	No	No
22	F	31	Cramps in calf muscles	10	2446	No	No
23	M	36	Difficulty getting out of a chair	20	11421	No	No
24	F	47	Poor at running	10	1940	No	Yes
25	M	18	No symptoms	Nil	3000	No	No
26	M	28	Cramps, myalgia	17	7000	Yes	No
27	M	51	Difficulty in running	14	1535	Yes	Yes
28	F	43	Cramps, myalgia	13	7260	No	No
29	F	42	Fatigability and difficulty climbing stairs	33	1528	Yes	No
30	F	40	Cramps, myalgia	6	3170	No	No
31	F	26	Cramps, myalgia	7	5000	No	No
32	F	38	Fatigability and weakness in lower limbs	16	5155	Yes	No
33	M	51	Fatigue with physical activities	37	n/a	Yes	No
34	F	58	Falling over	38	n/a	Yes	Yes
35	F	40	Difficulty getting out of a chair	32	n/a	No	No
36	M	64	Difficulty getting up from the floor	43	n/a		
37	M	45	Difficulty climbing stairs	34	n/a	No	No
38	M	21	Difficulty running and rising from a chair	10	n/a	Yes	Yes

The most frequent first symptoms were difficulty in climbing stairs (28%), difficulty in running (21.1%) and myalgia and cramps (21.1%). The creatine kinase was recorded in 32 of the 38 participants and ranged from 222 units/l to 23,858 units/l, with a mean of 5,142 units/l.

Respiratory involvement as reported by the patients was present in 21% (n = 8) of the cohort, however, 33.3% had a sitting FVC<75% predicted value for their height, and this rose to 69.0% for lying FVC. 16.6% had a>20% decrease in their FVC from sitting to lying and 33.3% had a 10–19% drop in their FVC from sitting to lying. 31% of the cohort that had a cardiomyopathy also had respiratory involvement (n = 5).

Cardiomyopathy was present in 16 of the 38 patients (42%) as documented on echocardiography results. 77% of those with cardiomyopathy were male and 23% female, the age range was 21–64 years, with a mean age of 44.4 years and 25% of those with a cardiomyopathy were in their 20's and all male.

### Analysis of T_1_w images

Pelvis, thigh and lower leg T_1_w images were analysed for both the control and patient group. The detailed distribution of grades amongst the 38 patients broken down per muscle group, along with the median grade for the muscle group is given in Table 2. In all 8 control subjects more than 95% of the muscles assessed were normal (grade 0) compared to the patient group, where less than 2% of the muscles assessed were normal and over 41% scored grades 3 or 4. There was a wide variation in median grades between muscle groups, with the thigh muscles generally more affected than those of the lower leg. Although the gluteus maximus muscle was not imaged quantitatively, the analysis of the T_1_w images revealed that 80% of the patients scored grade 3 or 4. The intraclass correlation coefficient between the two raters was 0.83, reflecting absolute agreement in scoring for 65% of assessments: the lowest agreement was for grade 2b, with 39% concurrence.

**Table 2:** Percentage of LGMD2I patients in each category of each semi-quantitative grade for individual muscle groups using the Mercuri et al. scale, with the median grade for each muscle.

Semi quantitative grade	0	1	2a	2b	3	4	Median grade
Muscle							
Gluteus Maximus (GM)	0	0	10.5	10.5	26.3	52.6	4
Biceps Femoris long head (BFLH)	0	2.6	10.5	10.5	13.2	63.2	4
Semitendinosus (ST)	0	5.3	13.2	10.5	31.6	39.5	3
Semimembranosus (SM)	0	0	18.4	26.3	18.4	36.8	3
Biceps Femoris short head (BFSH)	0	8.6	31.4	25.7	25.7	8.6	2b
Sartorius (SAR)	2.6	7.9	34.2	28.9	21.1	5.3	2b
Vastus Medialis (VM)	0	15.8	26.3	13.2	26.3	18.4	2b
Gracilis (GRAC)	2.6	10.5	42.1	13.2	21.1	10.5	2a
Vastus Lateralis (VL)	5.3	5.3	21.1	28.9	28.9	10.5	2b
Rectus Femoris (RF)	10.5	15.8	26.3	21.1	15.8	10.5	2a
Medial Gastrocnemius (MG)	5.3	7.9	18.4	23.7	21.1	23.7	2b
Lateral Gastrocnemius (LG)	2.6	18.4	18.4	23.7	28.9	7.9	2b
Peroneus Longus (PL)	0	18.4	26.3	34.2	18.4	2.6	2b
Soleus (SOL)	2.6	5.3	42.1	26.3	18.4	5.3	2a
Tibialis Anterior (TA)	7.9	34.2	47.4	10.5	0	0	2a

**Figure 1:**
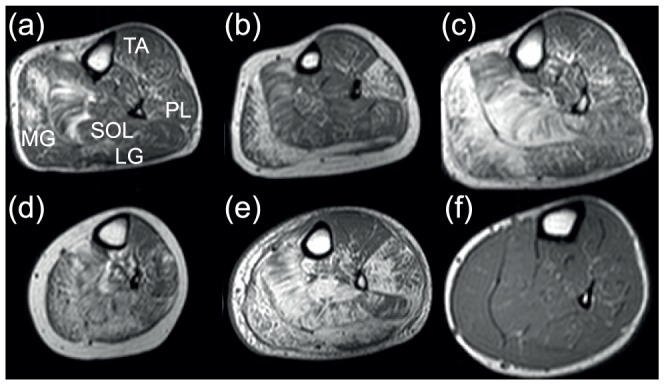
T1 weighted images of the lower leg in LGMD2I patients with increasing severity in pathology (a–e), and in a control subject (f). In image (a) there is involvement of medial gastrocnemius and soleus compared to image (e) where all muscles but tibialis anterior are severely affected. (TA =  tibialis anterior, SOL =  soleus, PL =  peroneus longus, LG =  lateral gastrocnemius, MG =  medial gastrocnemius).

In the lower leg, involvement of the gastrocnemii and soleus muscles was most noticeable with relative sparing of the tibialis anterior muscle until a late stage (Figure 1A–E). This was most striking in patients who had relatively severe involvement of the gastrocnemii with little change in the tibialis anterior muscles. The fat content and pattern seen in the lower leg muscles was distinct, with a variegated, striped appearance of the soleus (Figure 1A–C) and a lacy, reticular pattern in both lateral and medial gastrocnemius, commencing initially from the internal borders (Figure 1B & D). The peroneus longus muscle demonstrated ‘salt and pepper’ speckling (Figure 1B & E).

**Figure 2:**
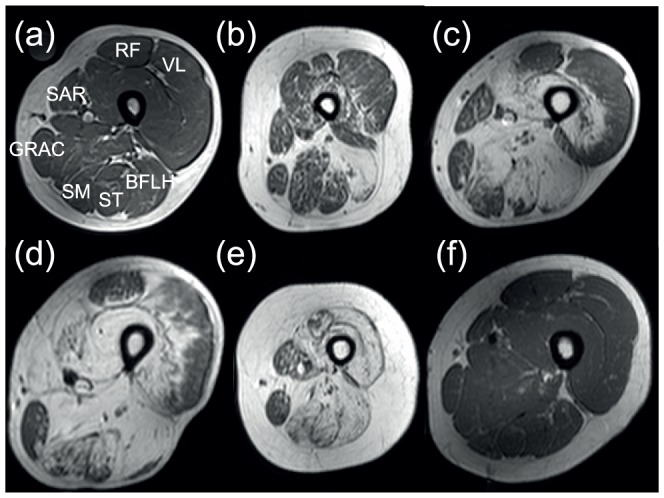
T1 weighted images of the thigh in LGMD2I patients with increasing stages of severity and fat content (a–e), and in a control subject (f). In (a) this is almost normal, however in (e) only sparing of gracilis and sartorius is seen. (VL =  vastus lateralis, SM =  semimembranosus, ST =  semitendinosus, BFLH =  biceps femoris long head, SAR =  sartorius, GRAC =  gracilis, RF =  rectus femoris).

In the thigh it has been reported that initially posterior involvement is seen, followed by a gradual increase in fat content and extension of pathological changes to involve the anterior thigh muscles as the disease progresses [18], [19]. In our cohort the biceps femoris (long head) muscle was most severely affected with the semimembranosus and the semitendinosus muscles next (Figure 2B–E). These muscles had a reticular pattern of involvement again from the more internal borders out to the periphery (Figure 2C). The vastus lateralis muscle was generally spared until late in the disease process. The vastus lateralis muscle demonstrated peripheral sparing (Figure 2D), a reverse of the pattern in Bethlem and Ullrich congenital myopathy [20]. The sartorius and gracilis muscles were relatively spared and had a stippled appearance with hypertrophy when affected (Figure 2A, B, C & E). The rectus femoris was also relatively spared with gross hypertrophy in clinically less severe participants (21.1% of study cohort) (Figure 2D). Atrophied recti were seen in patients with more extensive changes.

### Quantitative fat imaging

**Figure 3:**
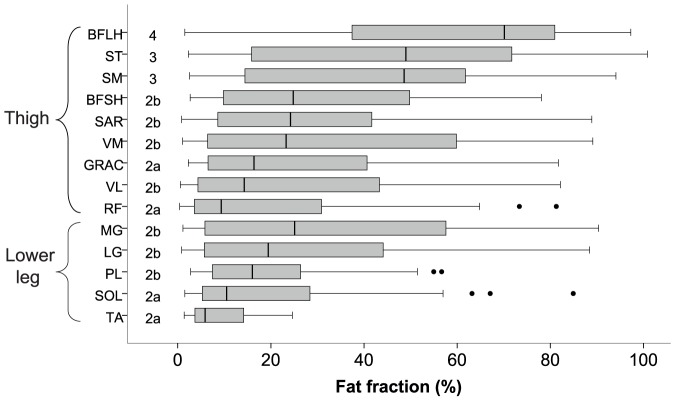
The grey bars illustrate the quantitative fat fractions for LGMD2I patients with median qualitative score at the left of the bars. The grey box indicates the lower and upper quartiles, with the median represented as a bar within the grey box and the stems the range (the outliers more than 1.5 interquartile range are marked separately as dots). (BFLH =  biceps femoris long head, ST =  semitendinosus, SM =  semimembranosus, BFSH =  biceps femoris short head, SAR =  sartorius, VM =  vastus medialis, GRAC =  gracilis, VL =  vastus lateralis, RF =  rectus femoris, MG =  medial gastrocnemius, LG =  lateral gastrocnemius, PL =  peroneus longus, SOL =  soleus, TA =  tibialis anterior).

Nine muscles at the mid thigh level and five muscles at the mid lower leg level, a total of 14 muscles per patient, were analysed in all 38 patients and 8 control subjects: Figure 3 summarises the quantitative results demonstrating that the degree of muscle pathology varied significantly from severe, as in the biceps femoris long head muscle (median fat fraction 69.7%), to very mild involvement in the tibialis anterior muscle (median fat fraction 5.9%). In the controls, the median fat fraction in all muscle groups ranged from 2.08% to 5.53%. Figure 3 also demonstrates how the severity of muscle pathology is spread with the most severely affected muscles being the biceps femoris long head muscle, followed by the semimembranosus and semitendinosus muscles, with rectus femoris and vastus lateralis muscles relatively spared in the thigh. In the lower leg, medial and lateral gastrocnemius muscles are more severely affected with tibialis anterior muscle relatively spared. Table 3 highlights that all muscles assessed quantitatively in the LGMD2I cohort were affected compared to the control group.

**Table 3:** The median values of fat fraction (%) in the patient group and the control group.

Muscle	Median LGMD2I	Median Control	P value
Biceps Femoris long head (BFLH)	69.7	3.9	0.0001
Semitendinosus (ST)	49.0	2.3	0.00001
Semimembranosus (SM)	48.6	2.9	0.0001
Biceps Femoris short head (BFSH)	25.5	3.2	0.001
Sartorius (SAR)	24.2	3.9	0.001
Vastus Medialis (VM)	23.3	3.0	0.01
Gracilis (GRAC)	16.4	3.0	0.0001
Vastus Lateralis (VL)	14.3	5.5	0.04
Rectus Femoris (RF)	9.4	3.3	0.03
Medial Gastrocnemius (MG)	25.1	2.2	0.001
Lateral Gastrocnemius (LG)	19.4	2.1	0.001
Peroneus Longus (PL)	16.0	4.9	0.01
Soleus (SOL)	10.5	3.0	0.001
Tibialis Anterior (TA)	5.9	2.8	0.02

The p values represent Mann-Whitney U test between patients and controls.

**Figure 4:**
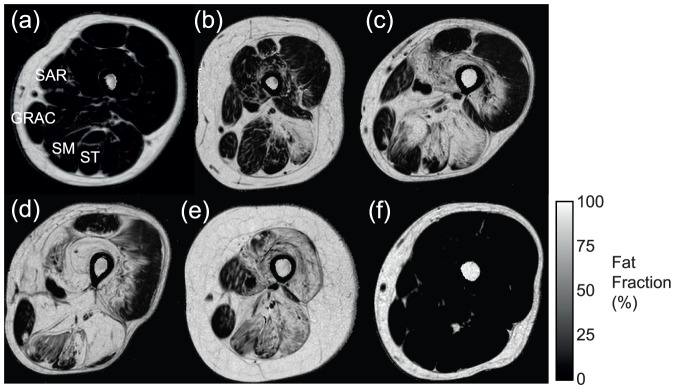
Quantitative fat images of the thigh in LGMD2I patients with increasing stages of severity and increasing fat fraction (a–e), and in a control subject (f). In (a) this is almost normal, however in (e) only sparing of gracilis (fat fraction 13.7%) and sartorius (24.0%) is seen. (SM =  semimembranosus, ST =  semitendinosus, GRAC =  gracilis, SAR =  sartorius).

**Figure 5:**
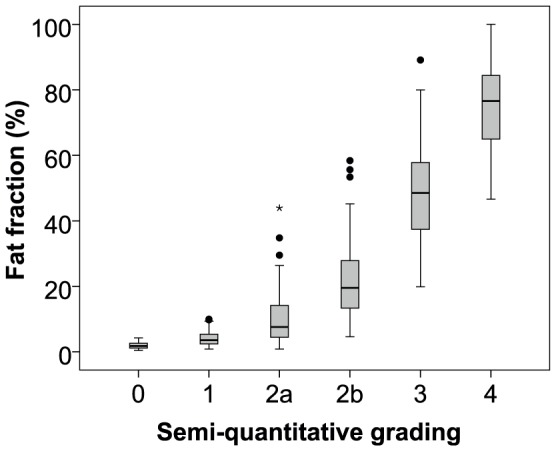
This figure compares the results of quantitative Dixon imaging with the semi-quantitative grading, demonstrating the wide range of fat fractions that can exist within the same grade (r_s_ = 0.87, p<0.01). The grey bars represent the middle 50% of the distribution between the upper and lower quartile and their corresponding fat fraction. The dots and stars represent outliers beyond 1.5 and 3.0 times the interquartile range beyond the upper quartile respectively.

The pattern of involvement is illustrated in the quantitative fat fraction images of Figure 4, with increasing severity in the thigh from an asymptomatic LGMD2I patient (a), with a semimembranosus (SM) fat fraction of 4.1%, to a more severely affected patient (e), with SM fat fraction of 51.8%. These are the same patients as Figure 2. Comparison of the T_1_w scoring and the quantitative fat imaging across all muscles showed correlation (r_s_ = 0.87 and p<0.01), but with significant overlap of fat fractions between qualitative grades (Figure 5). The inter-observer Bland-Altman analysis of quantitative fat imaging gave an inter-observer bias of 0.04% (no systematic bias) and an inter-observer repeatability coefficient of 1.43% (95% of comparisons fall within this range). The test-retest analyses with subject repositioning showed that the inter-scan repeatability coefficient in the fat fraction was 0.6% (with insignificant bias: 0.02%).There were some striking gender differences in muscle pathology in our LGMD2I cohort (Table 4), despite the fact that there was no difference in age, disease duration, or clinical severity as represented by 6MWD or FVC (Table S1 in File S1). In the female group there was diffuse involvement of both the gastrocnemii muscles; however, in the male group, the lateral gastrocnemius muscle had less fat infiltration. This contrasts with previous LGMD2I studies [18], [19], which reported diffuse and equal involvement of medial and lateral gastrocnemius compared to LGMD2A, where medial gastrocnemius is more involved than lateral gastrocnemius.

**Table 4:** Median values of fat fraction for the study cohort stratified by gender (19 males and 19 females).

Muscle	Male	Female
Semitendinosus (ST)	51.8	45.3
Semimembranosus (SM)	56.2*	28.2
Sartorius (SAR)	24.0	25.1
Gracilis (GRAC)	13.7**	25.0
Vastus Lateralis (VL)	11.2	18.2
Vastus Medialis (VM)	45.7§	18.9
Medial Gastrocnemius (MG)	22.2	28.0
Lateral Gastrocnemius (LG)	15.1†	33.7

It illustrates significant differences between the anterior thigh muscles in the male group and preferential sparing of the gracilis compared to sartorius. These features are not found in the female group.

^*^ p = 0.05 compared to male ST.

^**^ p = 0.01 compared to male SAR.

§ p<0.005 compared to male VL.

† p = 0.05 compared to male MG.

**Figure 6:**
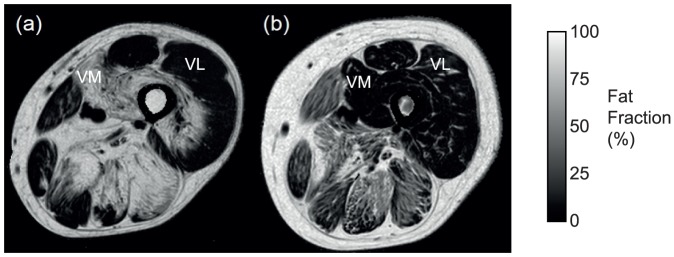
Comparison of the thigh muscles in (a) a male LGMD2I patient with more advanced pathology and increased fat content seen in the vastus medialis muscle (72.2%) and (b) preservation of the vastus medialis muscle (9.7%) of a female LGMD2I patient. (VM =  vastus medialis, VL =  vastus lateralis).

The hamstrings have previously been reported as more severely affected compared to the anterior thigh muscles [18], [19]. In the female subjects, there was a trend towards semimembranosus being spared relative to semitendinosus (median 28.2% compared with 47.3%). In males, these muscles were similarly affected, 56.2% and 51.8% respectively. The gracilis and sartorius muscles have previously been reported as being relatively and equally spared [18], [19]. In our male cohort, the gracilis muscle was more preserved compared to the sartorius muscle (Table 4, p = 0.01). In the female group, the gracilis and sartorius muscles were equally affected. The anterior thigh muscles also demonstrated gender differences; in the female subjects, the vastus lateralis and vastus medialis were affected similarly (18.2% and 18.9%), whilst in the male subjects there was a greater degree of fat infiltration, 45.7% in the vastus medialis compared to 11.2% in the vastus lateralis (p<0.005). This is shown in Figure 6 which compares similarly affected male and female patients with LGMD2I: preservation of the vastus medialis muscle seen in the female patient compared to the increased fat content and pathological changes seen in this muscle in the male patient.

### Functional correlations with MRI changes

**Figure 7:**
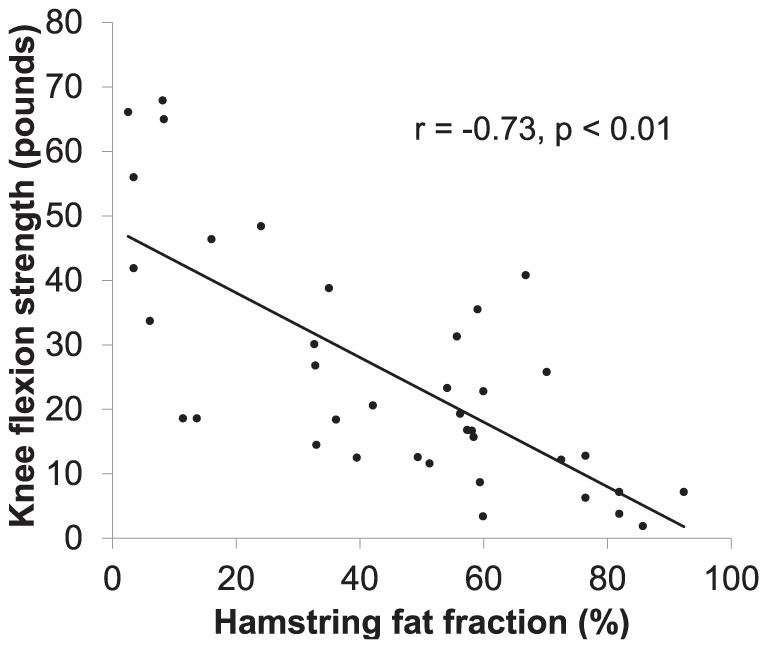
Strong correlation between knee flexion strength (in pounds) and hamstring average fat fraction, r = −.73 (p<0.01).

**Figure 8:**
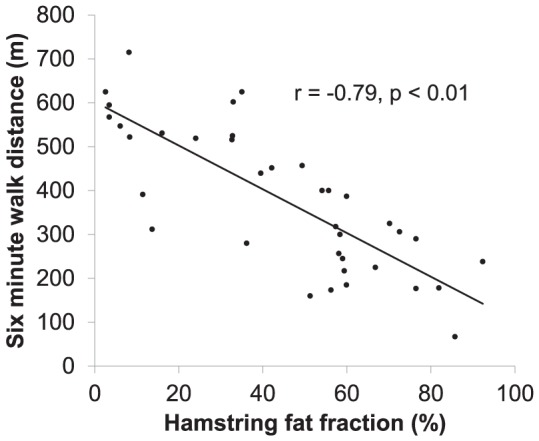
Strong correlation between 6 minute walk distance (6MWD) and hamstring average fat fraction, r = −.79 (p<0.01).

Strong correlations were demonstrated between myometry measures and fat infiltration in appropriately tested muscle groups. The ‘hamstring average fat fraction’ (the average of the combined fat fraction for semimembranosus, semitendinosus and biceps femoris muscle for each individual) correlated strongly with knee flexion strength r = −.73 (p<0.01) (**figure 7**) and the TUG test (r = .580, P<0.02). There was also strong correlation between ‘hamstring average fat fraction’ and the stair climb (r = .52, p<0.01), the stair descent (r = .46, p<0.01) and the 6MWD (r = −.79, p<0.01) (**figure 8**).

The vastus lateralis muscle (r = −0.75), vastus medialis muscle (r = −0.68) and the rectus femoris muscle fat fractions (r = −0.79) correlated strongly with knee extension strength. All were significant; p = 0.01. The ‘quadriceps average fat fraction’ (the average of the combined fat fraction for vastus lateralis, vastus medialis and rectus femoris muscle for each individual) strongly correlated with the stair climb (r = .718, p<0.01), the 6MWD (r = −.832, p<0.01), the timed chair rise (r = .743, p<0.01) and the TUG test (r = .753, p<0.01).

The 6MWD, used as an outcome measure in many clinical trials, strongly correlated with ‘average fat fraction’ (r = −0.80, p<0.01) (the average of the combined fat fraction of all 14 muscles analysed and calculated for each individual).

## Discussion

This is the largest reported cross-sectional MRI study (n = 38) of patients with LGMD2I due to the common mutation in the *FKRP* gene. Both traditional qualitative grading and quantitative fat imaging were applied to measure the differing degrees and patterns of muscle involvement and fat fractions. Attaining such a detailed natural history of a condition and its progression is vital as a basis for translating scientific research into a meaningful treatment. In LGMD2I disease progression and severity is heterogeneous and therefore objective reliable biomarkers are important. MRI is non-invasive, potentially objective and does not rely on patient effort compared to clinical and physical measures that are currently employed.

Previous reports of qualitative T_1_w MRI analysis in LGMD2I patients have demonstrated pathology preferentially in the posterior thigh muscles [5], [6], [18], [19], [21]. More specifically the changes seem to occur in the biceps femoris and internal adductor muscles first and with further disease progression to involve the rest of the hamstring muscles and to a lesser degree the vastus intermedius and lateralis muscles. Involvement of the vastus medialis and rectus femoris muscles has only been observed in those patients with advanced disease [19]. Our results confirmed the qualitative findings of these previous studies, but we have also demonstrated that in the male group there was a more severe and earlier involvement of the vastus medialis compared to the vastus lateralis muscle, not seen in the female group. This has not previously been documented in the published literature or reported in any other muscular dystrophy to date. In the lower leg, the gastrocnemius muscles have previously been reported as having diffuse and equal involvement [18], [19]. In our patient cohort, the male group demonstrated a predominantly medial involvement. This could mean that female patients with *FKRP* mutations phenotypically present differently to the male patients. This has been demonstrated in LGMD2L where female patients with *ANO5* mutations present and progress differently to male patients [22].

The use of MRI as a diagnostic tool has importance in many neuromuscular diseases; however, it does need to be used in conjunction with other assessments, such as clinical findings and muscle biopsy information. Whilst there are certain imaging patterns that appear to be pathognomonic, as in Bethlem myopathy and Ullrich myopathy [20], in LGMD2I there are similarities to other LGMDs, such as Calpainopathy (LGMD2A). In both these conditions, posterior thigh and lower leg involvement predominate, although it has been reported previously that there are differences in gastrocnemii muscle involvement, and that the vastus lateralis appears more spared in LGMD2A compared to LGMD2I. This, however, was not seen in our cohort of patients.

Comparison of the qualitative grades with the quantitative fat fractions showed that each grade corresponded to a very wide range of fat fractions, and whilst patterns of muscle pathology can be defined qualitatively, detecting small pathological changes in individual patients would be impossible using this system. In comparison, the quantitative method offers an increased discrimination and a more robust measure of muscle involvement for both cross-sectional and longitudinal studies. The inter-observer analysis suggests that these quantitative measures are highly observer independent.

This work also suggests that optimal target muscles for longitudinal assessment of pathological changes can be identified. The most suitable muscles are likely those demonstrating a range of fat fractions within the cohort, but are neither at the extremes of severity as with the biceps femoris (long head), semimembranosus and semitendinosus muscles, nor having limited involvement, such as the tibialis anterior muscle. Candidate muscles should also be easy to delineate on the MRI scans with well defined ROIs. The muscles that fulfil these criteria are the vastus lateralis, gracilis, rectus femoris and the gastrocnemii muscles. The inter-observer analysis showed that the results of quantitative analysis are highly objective.

A number of studies have addressed the more severe type of muscular dystrophy, Duchenne muscular dystrophy (DMD), highlighting both the disease natural history and its effect on muscles assessed by MRI [7], [21]. DMD is now a subject of clinical trials where, at present, therapeutic success is assessed by muscle biopsy and by functional outcomes, such as the six minute walk distance (6MWD). These tests, however, are either invasive or are highly dependent on patient cooperation. In comparison, MRI may provide markers that are both non-invasive, objective and as shown correlate highly with functional assessments, in particular the 6MWD, used in trials, and myometry strength testing.

Although this was the largest LGMD2I cohort studied with MRI to date, patient numbers remained relatively small, and future studies will benefit from widening the inclusion criteria to include both asymptomatic and paediatric patients. Measurements were also limited to quantification of the Dixon scans at one mid-point level in the lower leg and thigh. More sophisticated analyses may permit fat fraction determination at all levels in order to quantify the variability that is seen within each muscle. This may provide further insights into the process of muscle damage occurring in this condition. In addition to distributions of muscle involvement consistent with previous reports, we demonstrated a new observation of gender-dependent patterns. MRI fat fraction quantification provides objective measures of muscle pathology which are more definitive than the previous qualitative technique, and may provide a means to monitor previously undetectable small levels of change in longitudinal studies.

## Supporting Information

File S1 **Supporting information.** Figure S1, B_1_ inhomogeneity in conventional T_1_ weighted imaging. Figure S2, Analysis workflow for quantitative Dixon MRI and removal of B_1_ inhomogeneity. Table S1, Comparison of disease duration, six minute walk distance and forced vital capacity for male and female subjects.
(DOC)
