# Supplementary material for: Quantitative Magnetic Resonance Imaging in Limb-Girdle Muscular Dystrophy 2I: A Multinational Cross-Sectional Study
Source: PLoS One. 2014 Feb 28;9(2):e90377. doi: 10.1371/journal.pone.0090377 (PMC3938727; doi:10.1371/journal.pone.0090377)
Supplement: File S1 — Supporting information. Figure S1, B1 inhomogeneity in conventional T1 weighted imaging. Figure S2, Analysis workflow for quantitative Dixon MRI and removal of B1 inhomogeneity. Table S1, Comparison of disease duration, six minute walk distance and forced vital capacity for male and female subjects. (DOC) [file pone.0090377.s001.doc]

**Figure S1 :** B_1_ inhomogeneity in conventional T_1_ weighted imaging


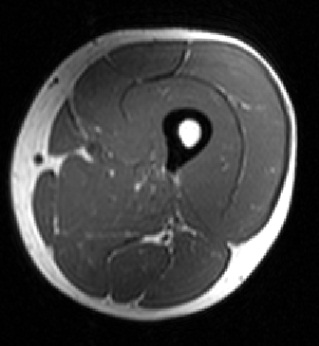


In standard T_1_ weighted imaging, the B_1_ (rf field) inhomogeneity that is always present means that the signal intensity of identical structures varies (such as that shown here in the subcutaneous fat, marked by arrows), so it is difficult to quantify muscle fat content. Usually the appearance is graded on a descriptive 6 point scale.

**Figure S2 :** Analysis workflow for quantitative Dixon MRI and removal of B_1_ inhomogeneity


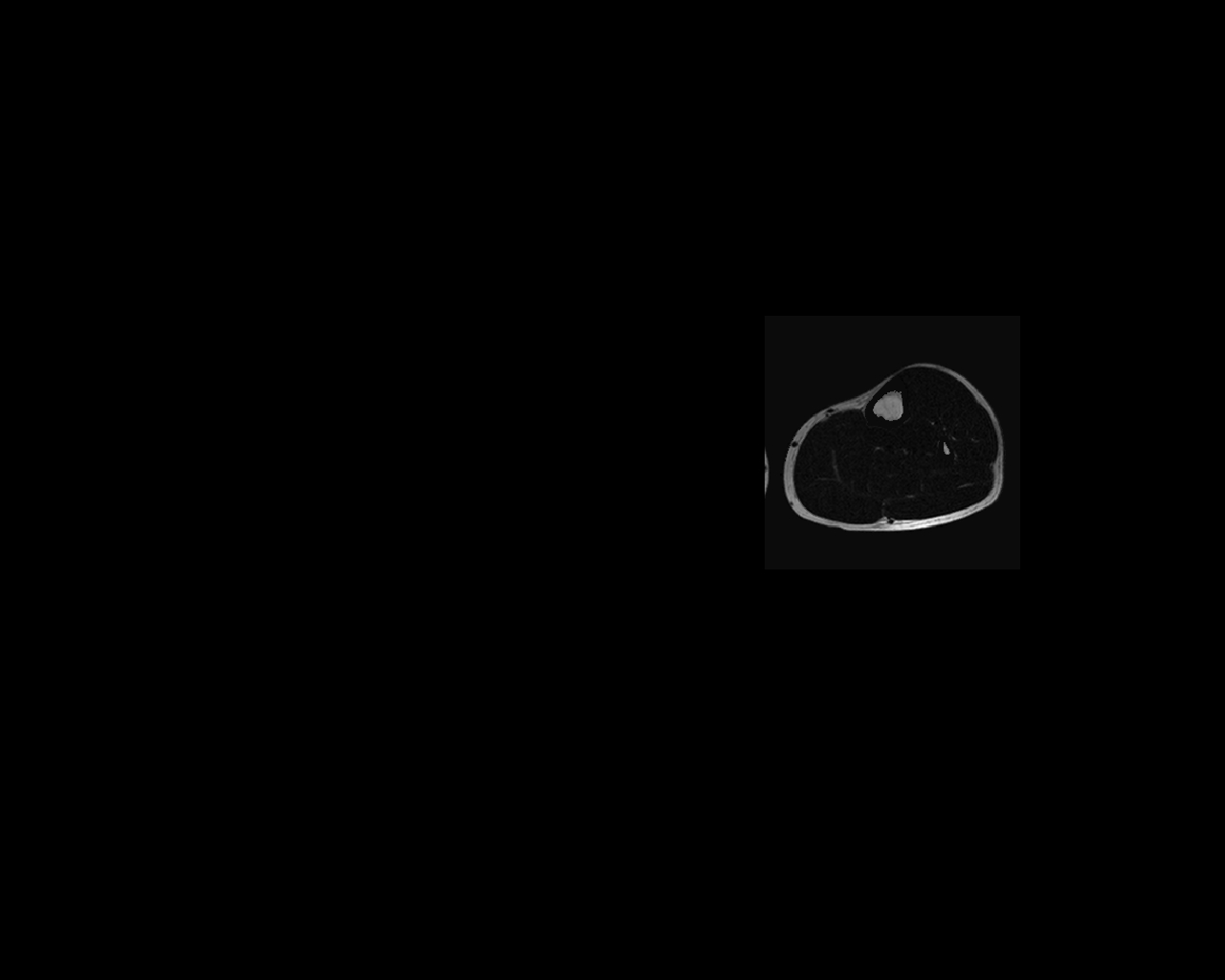

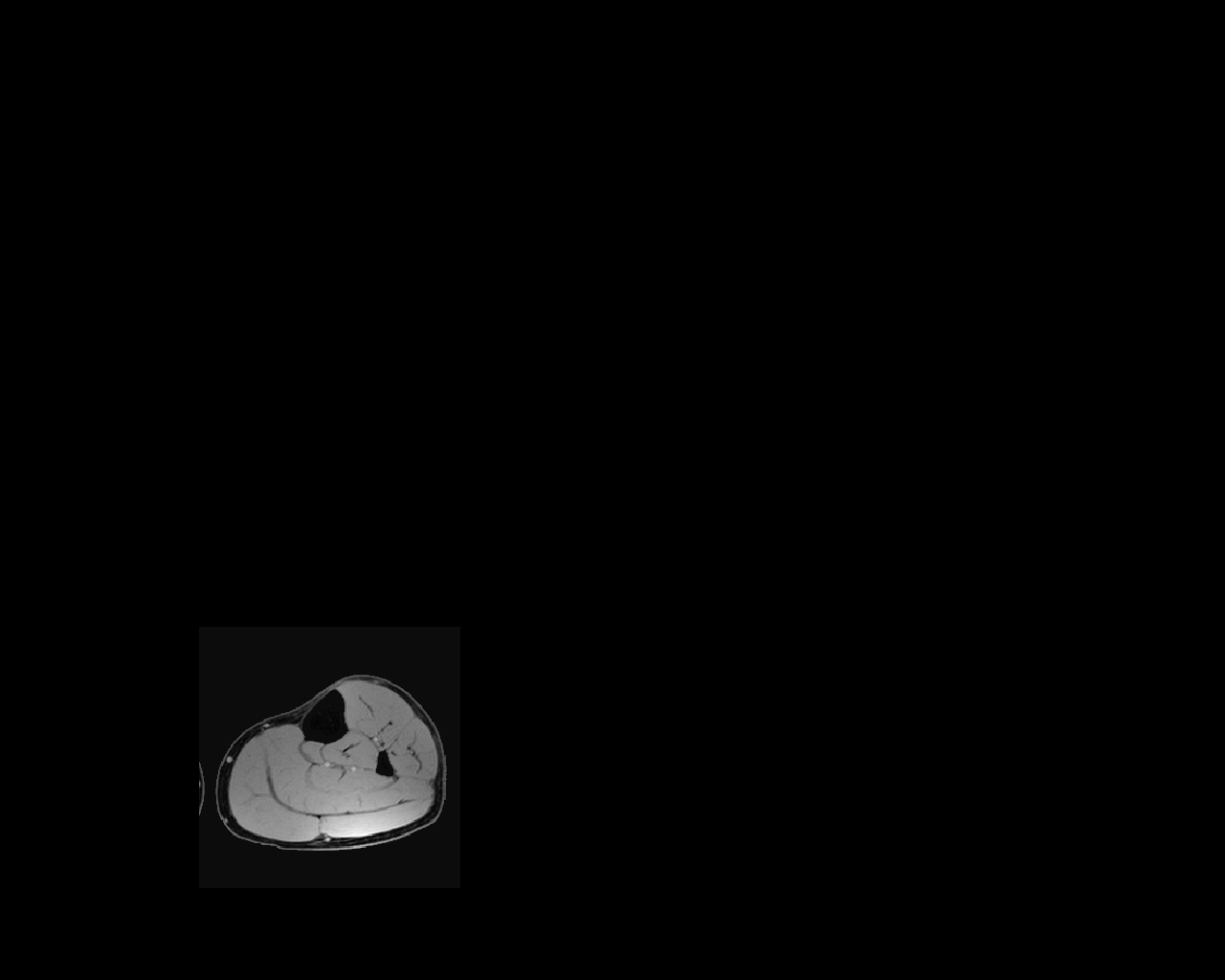

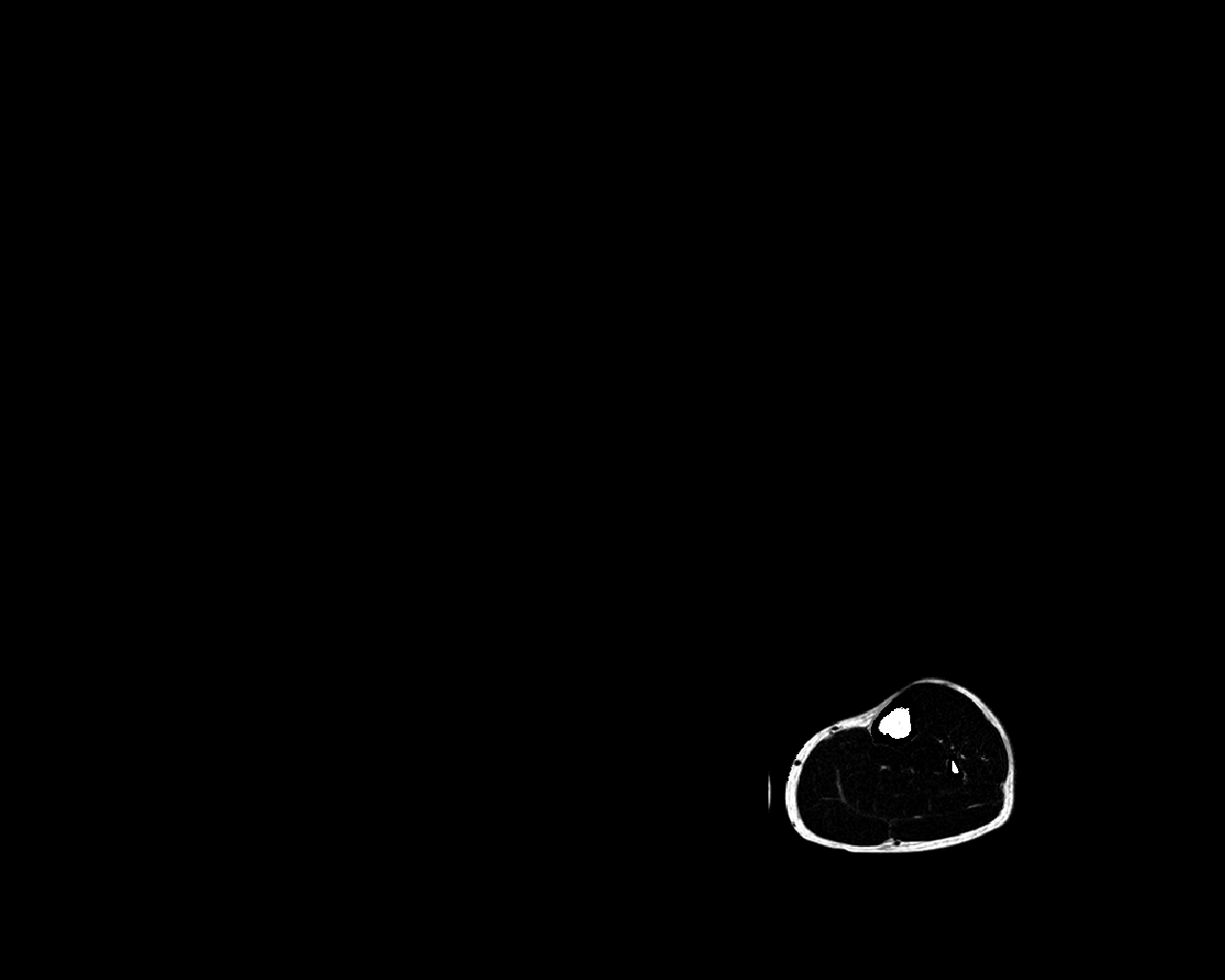

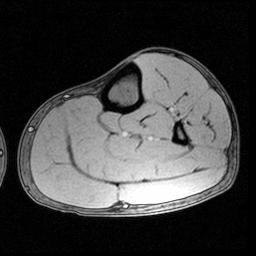

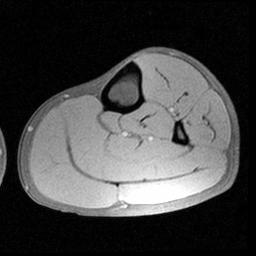

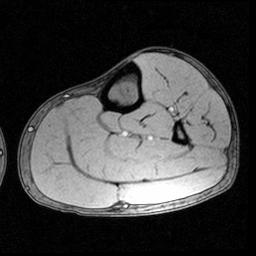


In the quantitative Dixon methods, 2 or 3 gradient echoes are acquired with different echo times which modulates the signals from fat and water, from left TE = 3.45,4.60,5.75ms. Note that in the first and third echoes fat and water signal cancels at the muscle/fat boundaries as the fat/water contributions are approx. 180^o^ out of phase, whereas it is additive in the second echo (in phase). These magnitude images still contain B_0_ and B_1_ inhomogeneity (brighter area marked by arrow). Phase data not shown.

The raw magnitude and phase data is analysed pixelwise^1,2^ to separate the water (*left*) and fat (*right*) contributions. B_0_ inhomogeneity has now been calculated and eliminated but the B_1_ inhomogeneity remains in these images (brighter area marked by arrow).

Since the B_1_ inhomogeneity is the same in both images, if we calculate the fat fraction (intensity in fat image over total intensity of fat+water), then the inhomogeneity cancels out and uniform structures have a uniform signal intensity from 0%-100% fat in the final image, which can then be measured by region-of-interest analysis. Some scanner implementations derive the same corrections from 2 echoes, as outlined in reference 13.

| **Table S1:** Comparison of disease duration, six minute walk distance and forced vital capacity for male and female subjects | | | |
| --- | --- | --- | --- |
|  | **Male** | **Female** | **P value** |
| N | 19 | 19 | ns |
| Age (years) | 40.9 ± 14.6 | 40.0 ± 9.8 | ns |
| Disease duration (years) | 16.5 ± 10.4 | 19.5 ± 8.7 | ns |
| Six Minute Walk Distance (m) | 357 ± 187 | 403 ± 140 | ns |
| Forced Vital Capacity (lying) | 71.8 ± 18.2 | 63.2 ± 13.6 | ns |
| Forced Vital Capacity (sitting) | 80.2 ± 13.0 | 75.9 ± 10.5 | ns |
